# Self-Powered Dye-Sensitized Solar-Cell-Based Synaptic Devices for Multi-Scale Time-Series Data Processing in Physical Reservoir Computing

**DOI:** 10.1021/acsami.4c11061

**Published:** 2024-10-28

**Authors:** Hiroaki Komatsu, Norika Hosoda, Takashi Ikuno

**Affiliations:** Department of Applied Electronics, Graduate School of Advanced Engineering, Tokyo University of Science, Katsushika, Tokyo 125-8585, Japan

**Keywords:** self-powered device, physical reservoir computing, synaptic device, artificial intelligence, dye-sensitized solar cell

## Abstract

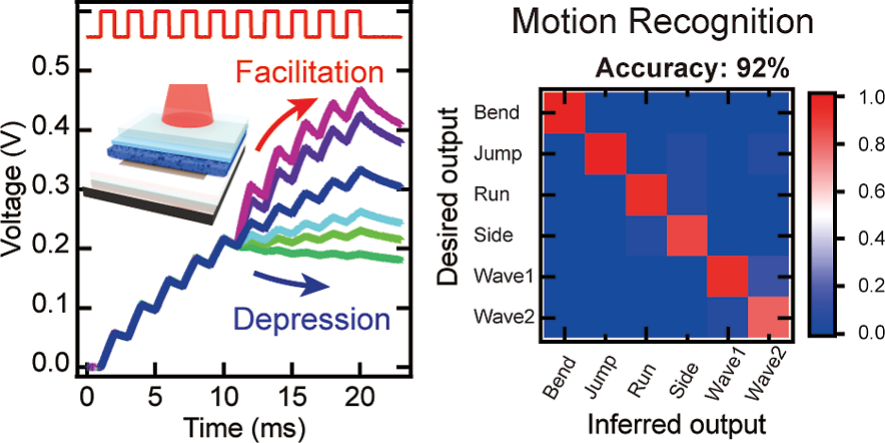

Physical reservoir computing (PRC) using synaptic devices has attracted attention as a promising edge artificial intelligence device. To handle time-series data on various time scales, it is necessary to fabricate devices with the desired time scale. In this study, we fabricated a dye-sensitized solar-cell-based synaptic device with controllable time constants by changing the light intensity. This device showed synaptic features, such as paired-pulse facilitation and paired-pulse depression, in response to light intensity. Moreover, we found that the high computational performance of the time-series data processing task was achieved by changing the light intensity, even when the input pulse width was varied. In addition, the fabricated device can be used for motion recognition tasks. This study paves the way for realizing multiple time-scale PRC.

## Introduction

With the rapid growth of sensor networks,^1^ new technologies can be developed to predict emergency events such as earthquakes,^2^ volcanic eruptions,^3^ heart attacks,^4^ and buried pipeline failures^5^ using artificial intelligence (AI). As the number of sensors increases, several challenges emerge, including higher network load, delayed data transfer, and increased power consumption on the server.^6^ To address these challenges, in-sensor edge AI devices that incorporate AI functionality within the sensor are in demand. To predict emergency events in advance, time-series data need to be processed by AI. Feed-forward artificial neural networks (ANNs), a common AI technology, are not inherently designed for time-series data processing.^7^ Therefore, reservoir computing (RC) has attracted considerable attention because it is specifically designed for time-series data processing. RC utilizes a reservoir layer with random coupling weights instead of a hidden layer in the feed-forward ANN, enabling time-series data to be handled with a lower power consumption. Among RC frameworks, physical reservoir computing (PRC), which uses physical devices as a reservoir layer, can integrate sensors and AI. Thus, PRC has garnered interest as a promising in-sensor edge AI technology. Thus, far, spintronic devices,^8,9^ memristor devices,^10,11^ ionic devices,^12,13^ and photonic devices^14,15^ have been reported to serve as reservoir layers in PRC.

The reservoir layer in PRC requires short-term memory (STM), nonlinearity, and high dimensionality.^16^ Artificial synapses, which mimic synapses utilizing physical devices, are gaining interest as a reservoir layer for the PRC because synaptic plasticity, a fundamental synaptic function, achieves STM. Specifically, the state of the reservoir depends on past and current inputs. Moreover, approximately 90% of the information received by the human brain comes from vision.^17^ Therefore, PRC with optoelectronic artificial synapses is expected to realize high recognition and real-time processing capabilities similar to those of the human visual system.

Thus, far, PRC with optoelectronic synaptic devices using various materials such as InGaZnO,^18^ cellulose-ZnO nanocomposites,^19^ and GaO_*x*_^20^ have been reported. However, these devices operate based on a photocurrent, resulting in high power consumption due to the need to apply a bias voltage. Therefore, self-powered photovoltaic devices are preferred devices for reducing power consumption because these devices can be driven by incident light input signals. Thus, far, PRC with self-powered optoelectronic devices has only utilized band bending mechanisms, and no systematic research has been conducted on PRC with photovoltaics-based artificial optoelectronic synapse devices using typical solar cells (i.e., silicon, copper indium gallium selenide, dye-sensitized, and perovskite solar cells). Lao et al. proposed a self-powered optoelectronic PRC system using band bending at the interface between semiconductors and insulators.^21^ This device showed 99.97% and 100% accuracy in face recognition and vehicle flow tasks, respectively. Additionally, the decay time constant of the device could be varied from 5.4 to 11.8 s by changing the number of light pulses. However, it was not possible to change the time constant significantly. Signals for monitoring infrastructure, the natural environment, and health conditions contain time-series data across various time scales. To handle these times-series data, the response time of the reservoir layer should be designed to match the time scale of the input data. Scholars have reported that dye-sensitized solar cells (DSCs) exhibit a time constant of the transient response at the open-circuit voltage (*V*_oc_) that depends on the light intensity (*P*).^22^ Therefore, the response time can be adjusted according to the input data without altering the device structure. Furthermore, a DSC requires almost zero operating energy and functions as an in-sensor device because it operates using the energy of the input light. In addition, the carrier transport kinetics in a DSC are complex and involve multiple electrochemical reactions.^23^ Consequently, DSCs can be expected to possess the nonlinearity required for the reservoir layer.^16^

In this study, we developed DSC-based synaptic devices to realize multiple-time scale PRC. Using a laser, we measured the transient voltage response as a function of *P*. This device exhibits synaptic features, such as paired-pulse facilitation (PPF) and paired-pulse depression (PPD). Moreover, it can be controlled by changing *P*. To evaluate the computational performance of our PRC system with DSC-based synaptic devices on various time scales, we conducted a STM task and a parity check (PC) task with different input pulse widths (*T*_p_). We found that *T*_p_ with maximum memory capacity in the STM and PC tasks varied depending on *P*. This result suggests that the operating time scale of our PRC system can be altered by changing *P*. This novel PRC system can be applied to the processing of time-series data across various time scales. As a demonstration, a motion recognition task was also performed. Our PRC system can recognize human actions with high accuracy and can be used in intelligent camera applications in the future.

## Results and Discussion

**Figure 1:**
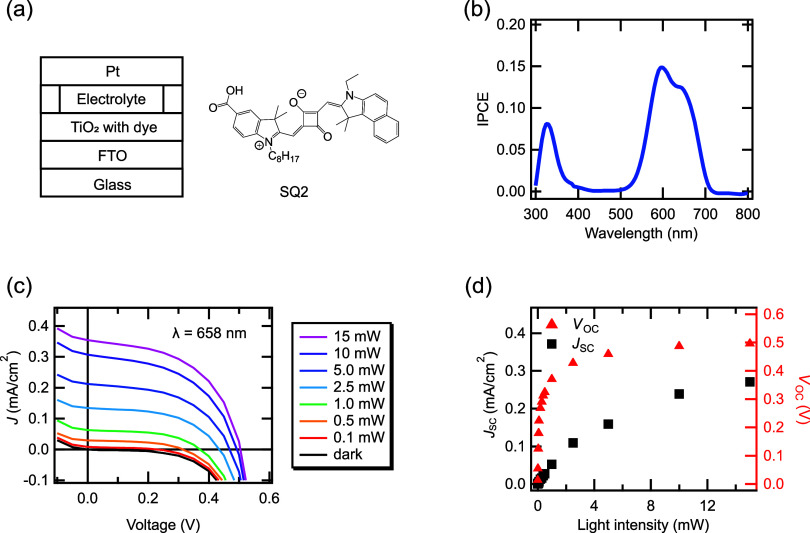
Fundamental characteristics of the fabricated DSC. (a) Device structure of the DSC and molecule structure of SQ2. (b) Incident photon-to-current conversion efficiency of the DSC. (c) *J*–*V* curves of the DSCs (λ: 658 nm). (d) *V*_oc_ and *J*_sc_ as a function of the incident light intensity, *P*.

We fabricated DSCs with squarylium derivative-based dyes (SQ2) that exhibit an absorption range of 550–700 nm. Figure 1a depicts the device and molecular structures of the SQ2. Figure 1b presents the incident photon-to-current conversion efficiency (IPCE) for the DSC. The spectrum shows two absorption ranges: 305–365 and 530–700 nm. The former matched the band edge absorption of TiO_2_ (3.2 eV),^24^ whereas the latter was consistent with the absorption spectrum of SQ2. Then, a 658 nm laser was employed as the input light to distinguish between the injected electrons from the dye and the excited electrons in TiO_2_. Figure 1c displays the *J*–*V* curve as a function of the *P*. As expected, *V*_oc_ and the short-circuit current (*J*_sc_) increase with *P*. Figure 1d presents *V*_oc_ and *J*_sc_ as functions of *P*.

**Figure 2:**
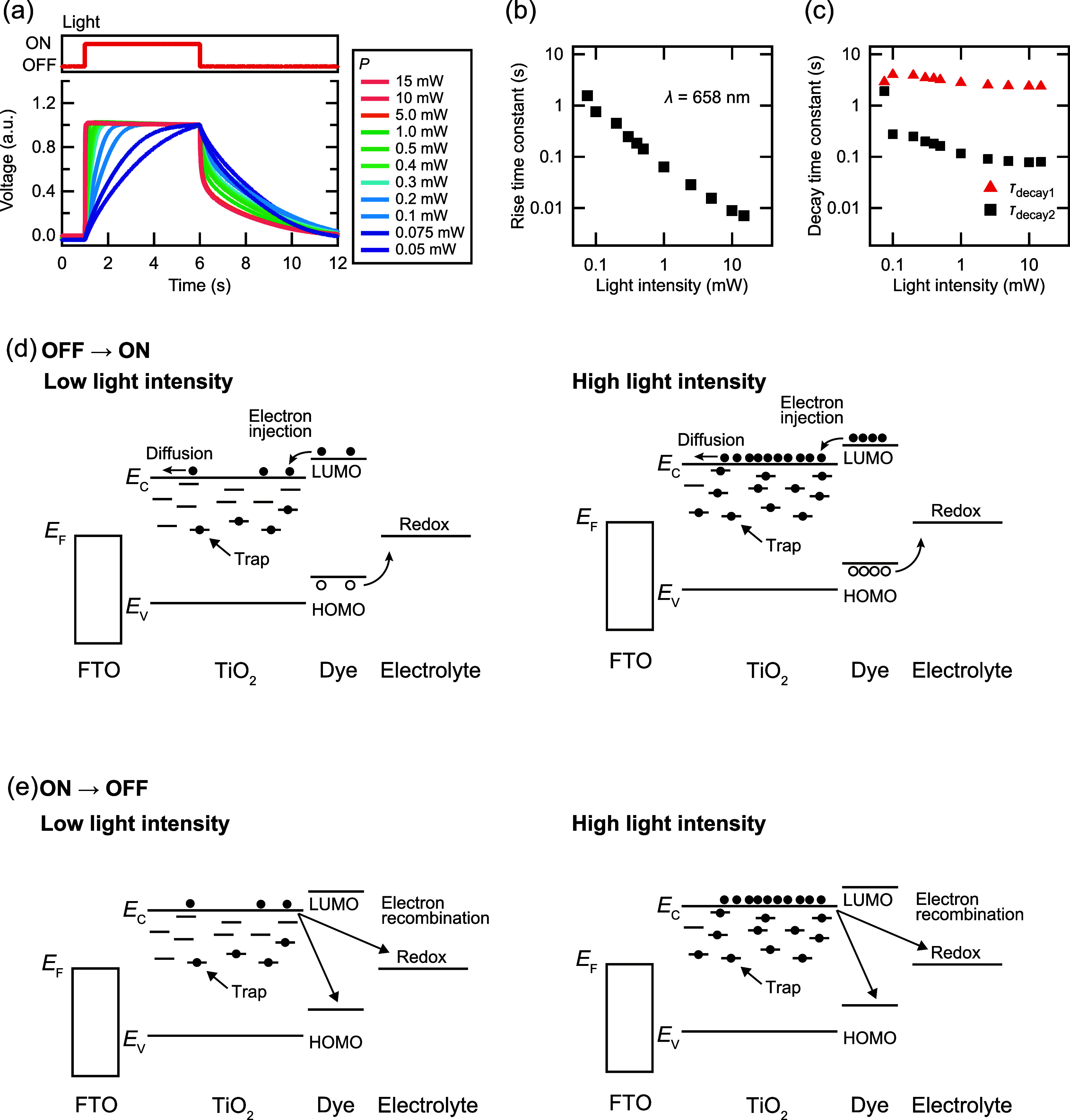
Transient voltage responses. (a) Normalized transient voltage responses as a function of *P*. (b,c) Rise time constant and decay time constant as a function of *P*. (d) Band diagram of TiO_2_ at high and low *P* levels when light irradiation starts. (e) Band diagram of TiO_2_ at high and low *P* levels when the light irradiation at a steady state is stopped.

We investigated the transient voltage response with respect to *P*, as illustrated in Figure 2a. At low *P*, such as 0.05 mW, it takes 5 s to reach *V*_oc_. By contrast, at high *P*, such as 15 mW, it takes 45 ms to reach *V*_oc_. To further explore the effect of *P* on the transient voltage response, we estimated the average rise time (τ_rise_) and decay time (τ_decay_) constants, as shown in Figure 2b,c. The τ_rise_ and τ_decay_ curves were fitted using single and double exponential functions, respectively. When *P* increases from 0.1 to 10 mW, τ_rise_ dramatically decreases from 0.75 s to 8.9 ms. Moreover, τ_decay_ decreases with *P* in the range of 0.1–1 mW. Above 1 mW, τ_decay_ remains constant within the 1–10 mW range, regardless of *P*. This feature of varying time constants with *P* is unique among solar cells. The typical transient voltage response of polycrystalline Si solar cells does not change with *P* (Figure S1). The rise time constant remains constant regardless of *P*, in contrast to DSC (Figure S2).

For DSCs, scholars have reported that τ_rise_ decreases with increasing *P*.^22^ This phenomenon can be attributed to the fact that the diffusion coefficient (*D*_n_) of free electrons in TiO_2_ depends on *P*. In other words, *D*_n_ depends on the electron density (*n*_c_) in the conduction band of TiO_2_. Carrier transport in the TiO_2_ layer in DSCs can be interpreted by using the multiple trapping (MT) model. This model has been adopted for nanostructured semiconductors, drawing from extensive experience with disordered semiconductors.^25^ By applying the MT model, solving the continuity equation for the electrons injected into the TiO_2_ layer, and considering trapping and detrapping processes, we obtain *D*_n_ as
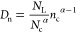
1where *N*_L_ is the total density of localized sites (per unit volume), *N*_C_ is the effective density of the conduction band states, and α is a parameter that is obtained by dividing the temperature (*T*) by the tailing parameter *T*_0_ (α = *T*/*T*_0_). We assumed that traps had an exponential distribution. *D*_n_ is also known to depend on *n*_c_. Specifically, at high *P*, high diffusion coefficients are obtained owing to an increase in *n*_c_, which can shorten τ_rise_. Figure 2d depicts the energy diagram for a DSC when light irradiation starts at low and high *P*, respectively. Upon light irradiation, electrons are excited from the highest occupied molecular orbital level to the lowest unoccupied molecules orbital level in the dye. Then, the electrons are injected into TiO_2_. At low *P*, *D*_n_ is reduced because many electrons are trapped in comparison to the conduction band electrons. Thus, τ_rise_ is longer at low *P*. By contrast, *D*_n_ increases at high *P* because fewer electrons are trapped than conduction band electrons. Therefore, τ_rise_ is shorter at high *P*.

When light irradiation at a steady state is stopped, the voltage decays with time. The electron lifetime (τ_*n*_) can be expressed as
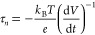
2where *V* is the open-circuit voltage decay curve of DSC and *k*_B_*T*/*e* is the thermal energy, in which *k*_B_ and *e* are the Boltzmann constant and elementary charge, respectively.^26^ Therefore, τ_decay_ depends on τ_*n*_, which governs the recombination process. No electron recombination occurs in TiO_2_ owing to the absence of hole carriers. Possible recombination processes include either transferring electrons to the oxidized dye or transferring electrons to the acceptor in the electrolyte, as depicted in Figure 2e. Numerous studies have assumed that oxidized molecules are rapidly regenerated, resulting in recombination occurring only with the acceptor in the electrolyte.^23^ Salafsky et al. reported that the recombination rate with the acceptor ranges from a few hundred milliseconds to a few seconds.^27^ In addition, Huang et al. found that the reaction with I_2_^–^ can be rate-limiting.^28^ Consequently, in our work, the negligible change shown by τ_decay_ in response to *P* can be attributed to the rate-limiting reaction with I_3_^–^. Considering this concept, the voltage response time of DSC can be altered by changing the *P*. This characteristic suggests that the response times of DSC-based synaptic devices and in PRC applications can be controlled by simply adjusting the *P*.

**Figure 3:**
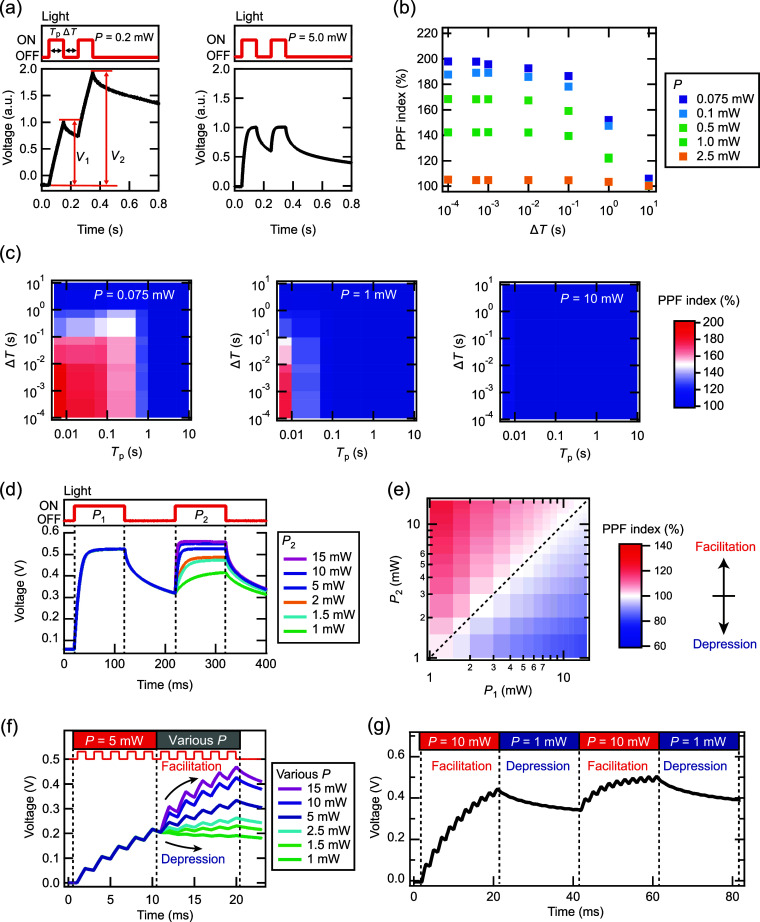
Synaptic characteristics in the DSC. (a) Transient voltage responses induced by two optical pulses with high and low *P* (λ: 658 nm, *T*_p_: 100 ms, Δ*T*: 100 ms, and *P*: 0.2, 5.0 mW). (b) PPF index as a function of Δ*T* (*T*_p_: 100 ms, *P*: 0.075–2.5 mW). (c) *T*_p_ dependence of PPF index as functions of *P* and Δ*T*. (d) Transient voltage response induced by two optical pulses with different *P* values (λ: 658 nm, *T*_p_: 100 ms, Δ*T*: 100 ms, *P*_1_: 5 mW, and *P*_2_: 1–15 mW). (e) PPF index map for *P*_1_ with various *P*_2_ (λ: 658 nm, *T*_p_: 100 ms, *P*_1_: 1–15 mW, and *P*_2_: 1–15 mW). (f) Transient voltage responses induced by 10 optical pulses with various *P* values (*T*_p_: 1 ms, *P*_1_: 1–15 mW). (g) Switching facilitation and depression of the DSC by changing *P* (*P*: 1, 10 mW).

Next, we investigated the effect of *P* on the synaptic features of the DSC-based synaptic devices. Short-term plasticity is a fundamental synaptic characteristic that is believed to be closely related to the ability to decode temporal information.^29^ Among them, PPF and PPD, which are facilitation or depression induced by two successive pulses, are typically utilized to evaluate the characteristics of synaptic devices.^30^Figure 3a shows a typical transient voltage response induced by low and high *P* (λ: 658 nm, *T*_p_: 100 ms, and *P*: 0.2, 5.0 mW). At low *P*, the voltage increases with successive light irradiation. By contrast, at high *P*, the voltage was saturated with the first pulse and did not increase with the second light irradiation. This is because short time constants with high *P* values resulted in saturation during the first pulse. Once saturated with the first pulse, there is no room to increase with the second pulse. Therefore, PPF is expected to occur when *T*_p_ is less than time constant. To investigate the PPF in the DSC-based synaptic device, we measured the PPF index, which is defined as *V*_2_/*V*_1_ × 100, as displayed in Figure 3a. Figure 3b shows the PPF index as a function of Δ*T* with various *P* values. The PPF index increases with decreasing Δ*T*. When Δ*T* is fixed, the PPF index increases with decreasing *P*, reaching a maximum of 223% (*P*: 0.075 mW, Δ*T*: 10^–4^ s). Figure 3c shows the average PPF index as a function of *T*_p_ and Δ*T* with various *P* values. Besides Δ*T*, *T*_p_ also influences the PPF index. Interestingly, the PPF index distribution, which exhibits a high PPF index above 100%, shifts with the changes in *P*. For example, when *P* = 10 mW, no facilitation takes place, leading to a 100% PPF index at *T*_p_ = 0.1 s. However, upon reduction of *P* to 0.075 mW, facilitation occurs, resulting in a 175% PPF index at *T*_p_ = 0.1 s. When *T*_p_ is in the range of subseconds to tens of milliseconds, no facilitation occurs at high *P*; however, facilitation takes place when *P* is reduced. This result indicates that the response time, which exhibits synaptic features, can be modified by adjusting *P*.

To achieve depression, we changed *P* at the first (*P*_1_) and second pulses (*P*_2_). Figure 3d illustrates the transient voltage response at a constant *P*_1_ with various *P*_2_ values (*P*_1_: 5 mW, *P*_2_: 1–15 mW). When *P*_2_ is smaller than *P*_1_ (e.g., *P*_2_ = 1–2 mW), *V*_2_ is lower than *V*_1_. Therefore, PPD is achieved at *P*_2_ < *P*_1_. By contrast, *V*_2_ is greater than *V*_1_ (e.g., *P*_2_ = 5–15 mW), which implies that PPF is achieved at *P*_2_ > *P*_1_. Figure 3e shows the average PPF index map for *P*_1_ with various *P*_2_ values (λ: 658 nm, *T*_p_: 100 ms, *P*_1_: 1–15 mW, and *P*_2_: 1–15 mW). In addition, the PPF index is approximately 100% when *P*_1_ and *P*_2_ have comparable values. When *P*_2_ > *P*_1_, the PPF index exceeds 100%, indicating facilitation. By contrast, when *P*_2_ < *P*_1_, the PPF index is less than 100%, suggesting that depression occurs.

Figure 3f displays the transient voltage response induced by 10 light pulses. The first five pulses were irradiated at *P* = 5 mW, and the remaining five pulses were irradiated at various *P* values. Facilitation or depression occurs in response to *P*. Figure 3g shows the transient voltage response when *P* is changed every 10 pulses. Depression and facilitation occur in response to *P*. Therefore, the accumulation and forgetting of time-series changes in *P* are reflected as variations in the voltage. This feature is known as fading memory, which is a desirable characteristic for the reservoir layer in the PRC. Consequently, DSC-based synaptic devices can be applied to the reservoir layer in the PRC.

**Figure 4:**
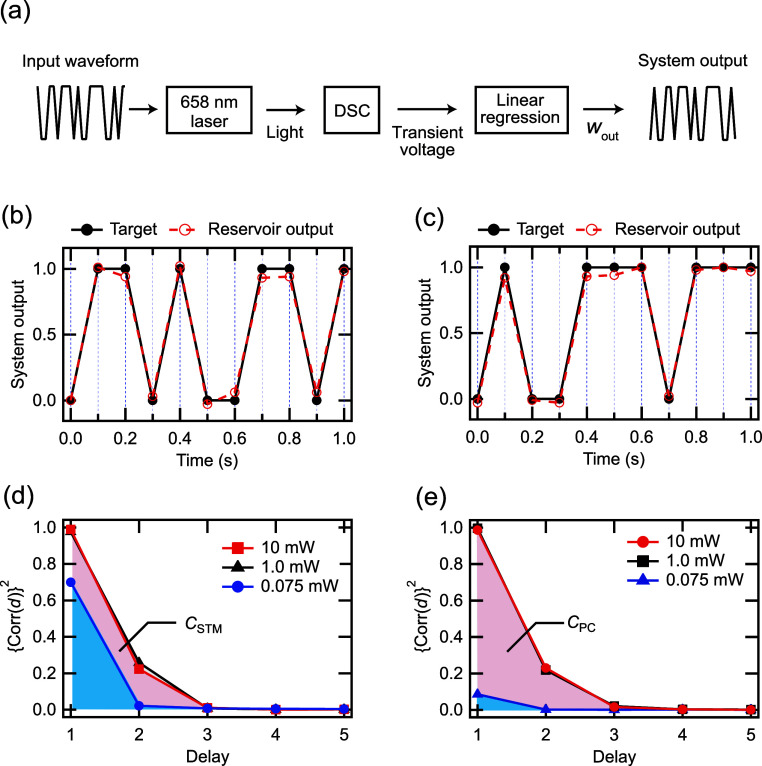
Time-series data processing task. (a) Operating scheme of the time-series data processing task with a DSC-based synaptic device. (b,c) System output of the STM and PC tasks (*d*: 1, *T*_p_: 100 ms, *P*: 1 mW, and number of virtual nodes: 125). (d,e) Forgetting curves of the STM and PC tasks with various *P* (*d*: 1, *T*_p_: 100 ms, *P*: 0.075–10 mW, and number of virtual nodes: 125).

To verify the feasibility of our DSC-based synaptic device for PRC, a standard benchmark task for time-series processing was performed. We used STM and PC tasks.^31,32^ The STM task, which assesses the ability to reproduce past input, is used as a benchmark task to evaluate short-term memorability. The PC task, which assesses the ability to reproduce exclusive or past input, is utilized as a benchmark task to evaluate nonlinearity. The operating scheme of a PRC with a DSC-based synaptic device is illustrated in Figure 4a. Initially, random binary input waveforms are converted into light pulses. Then, light pulses are irradiated on the device using a laser. The transient voltage is measured and fed into the NN to calculate the output weight (*W*_out_). The target waveform is then calculated using *W*_out_. For each task, the input data (*y*_input_) and target data (*y*_target_) are described by the following equations
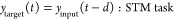
3
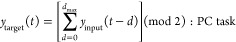
4

Figure 4b,c shows the system outputs for the STM and PC tasks for a DSC-based synaptic device (*d*: 1, *T*_p_: 100 ms, *P*: 1 mW, and virtual node: 125). One can estimate the memory capacity (*C*) using
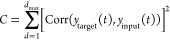
5where “corr” denotes the correlation coefficient. A larger *C* value indicates that more past input is retained or that more nonlinearities are present in the reservoir. Figure 4d,e displays the square of the correlation coefficient as a function of delay for the STM and PC tasks with various *P* (*d*: 1, *T*_p_: 100 ms, *P*: 0.075–10 mW; and virtual node: 125). The PRC system with DSC-based synaptic device exhibits maximum values of *C* in the STM task (*C*_STM_) and PC task (*C*_PC_) for 1.31 and 1.13, respectively. Compared to previous literature on various physical reservoirs, our *C*_STM_ values are smaller than those reported values (*C*_STM_: 1–35).^19,33−38^ Meanwhile, the *C*_PC_ is comparable to those reported values (*C*_STM_: 0.4–1.7).^19,35,36^ In addition, to verify the capability of a DSC-based synaptic device for PRC, STM and PC tasks were performed with the input signal itself. The PRC system without DSC-based synaptic device exhibits nearly zero value of *C*_STM_ and *C*_PC_, as shown in Figure S3. We found that *C*_STM_ and *C*_PC_ are higher when the light input has medium to high intensities compared to low intensity. This indicates that longer time constants of the device do not necessarily lead to higher STM or nonlinearity. Previous studies have shown that high *C*_STM_ is exhibited when the time constants of the device and *T*_p_ are comparable.^33,39^ Therefore, we investigated the correlation between the pulse width of light at each irradiation intensity and both *C*_STM_ and *C*_PC_.

**Figure 5:**
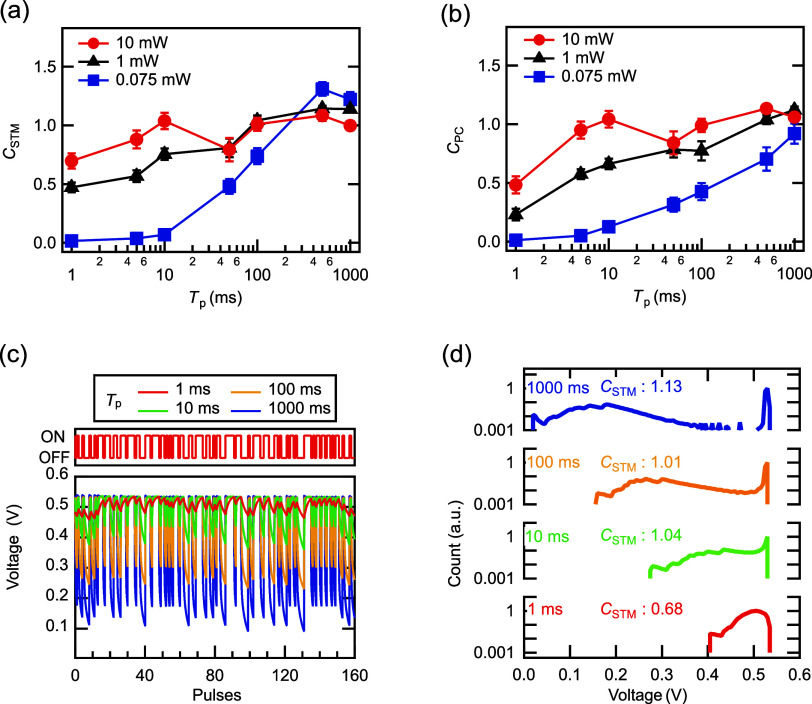
Time-series data processing task as a function of *T*_p_ with various *P*. Error bar represents standard error of the mean. (a) Average *C*_STM_ as a function of *T*_p_. (b) Average *C*_PC_ as a function of *T*_p_. (c) Transient voltage response induced by random binary pulses with various *T*_p_ (*P*: 10 mW, *T*_p_: 1–1000 ms). (d) Histogram of the transient voltage response with various *T*_p_ (*P*: 10 mW, *T*_p_: 1–1000 ms).

We fabricated eight samples and characterized their *C*_STM_ values and *C*_PC_. Figure 5a,b shows average *C*_STM_ and *C*_PC_ as a function of *T*_p_ at each light intensity, respectively. At a light intensity of 0.075 mW, *C*_STM_ reached a maximum value of 1.31 at *T*_p_ = 500 ms. While the value remained almost unchanged for *T*_p_ longer than 500 ms, we observed the steep decline in *C*_STM_ for *T*_p_ shorter than 500 ms. At light intensities of 1 mW or higher, relatively high *C*_STM_ was maintained even in the region where *T*_p_ was less than 100 ms. In other words, within the time range of this graph, with increasing light intensity, the range of pulse widths that yield high *C*_STM_ values expands. *C*_PC_ showed a similar trend. As the irradiation intensity increased, the range of pulse widths that exhibited high *C*_PC_ values also expanded. Within the time range of this graph, it was observed that higher irradiation intensities resulted in higher *C*_PC_ values (with a maximum of 1.13 at *P* = 10 mW).

We conducted a more detailed investigation to understand why the memory capacity changes with respect to *T*_p_. Figure 5c shows the output voltage in response to random input pulses (*T*_p_ = 1–1000 ms) used to characterize the STM task. The light intensity was 10 mW. It was observed that the range of output voltage varies depending on *T*_p_. Figure 5d displays the histogram of the output voltage. The maximum value of all the data represents the *V*_oc_ of the DSC. The results showed that the longer the *T*_p_, the wider the range of the output voltage. Looking into the details, it appears that when *T*_p_ is smaller than the time constant of the device (τ_rise_ = 8.9 ms, and τ_decay_ = 2.3 and 79 ms at *P* = 10 mW), the range of output voltage becomes narrower. A wider output voltage range corresponds to a higher *C*_STM_. The reason might be that the wide range of output voltage would increase the variation in the values of the virtual nodes, resulting in an improvement in reproducing past input. These results suggest that a device design capable of achieving a wide range of output voltage across various light irradiation intensities and time scales is crucial for realizing high memory capacity. In applications where the irradiation intensity is fixed, a design that corresponds to the time scale of the incident light will be required. Additionally, from another perspective, when the pulse width and light intensity of the input light signal change simultaneously, the output voltage profile becomes non-exponential, which suggests that a physical reservoir with high nonlinearity can be constructed.

The fabricated DSC-based synaptic device exhibits the unique capability of output voltage values based on the accumulation and decay of time-series variations in *P*. This characteristic suggests that the device may be well-suited for processing time-series data related to *P*. To validate this, we conducted a motion recognition task using time-series changes in *P* as an input. The objective of this task was to classify the actions depicted in a video. In this experiment, a single DSC was utilized.

**Figure 6:**
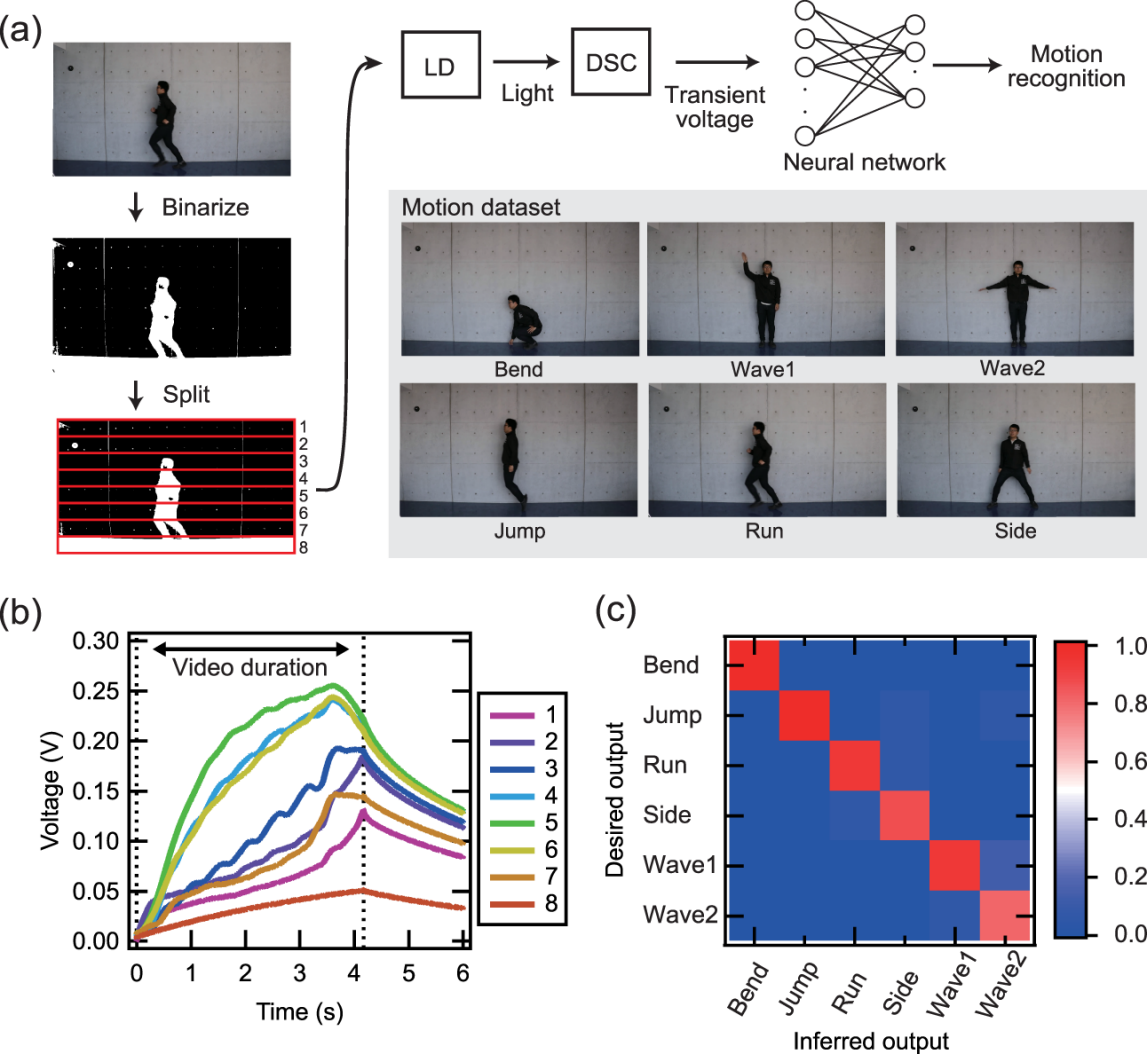
Demonstration of motion recognition task using a PRC system with the DSC-based synaptic device. (a) Schematic diagram of the motion recognition task using the DSC. (b) Transient voltage response of the DSC output with eight divided motion movies (jump). (c) Confusion matrix of the motion recognition tasks.

Figure 6a shows a schematic diagram of the motion recognition task using a PRC system using a DSC-based synaptic device. Human motions were captured by a camera and then binarized. Next, the video was split into eight parts, and the time-series data of the average luminance [*L*(*t*)] for each strip were obtained. *L*(*t*) was converted into *P* [*P*(*t*)] and then input into the DSC through a laser. The time scale of human motion is on the order of subseconds to seconds. Therefore, *P* was set to 0.1–1 mW with subsecond to second time constants. The next input was applied after waiting for complete relaxation of the voltage. The obtained voltage values were fed into a NN for motion recognition. Figure 6b displays the transient voltage responses with eight divided motion videos (jump). Figure 6c shows the confusion matrix of the motion recognition task. All actions (i.e., bend, jump, run, side, wave1, and wave2) are distinguished with over 80% accuracy. The total accuracy is 92%, indicating that motion recognition is successfully achieved by using the fabricated device. For comparison, the results of the motion recognition task in different machine-learning conditions are presented (Figure S4).

To verify the effectiveness of the fabricated device as part of a PRC system, we also conducted a motion recognition task without a DSC-based synaptic device. Figure S5 shows the results of this task, highlighting the performance comparison. The calculated accuracy of the PRC system without DSC-based synaptic device saturated at 17%. This result underscores the potential of DSC-based synaptic devices within the PRC system. We believe that our device, which has significantly fewer pixels than a modern camera, could achieve recognition tasks comparable to the human visual system by utilizing the synaptic responses of the DSC-based synaptic device. For this demonstration, we used laser light as an ideal light source due to the limited amount of training data. For practical applications, environmental light could serve as a light source if the volume of training data is increased. In addition, our device can be configured in a two-dimensional array to provide spatial resolution, enabling the realization of advanced neuromorphic vision systems.

## Conclusions

In this study, we fabricated DSC-based synaptic devices to achieve PRC. By adjusting *P*, these devices can significantly control τ_rise_ from 8.9 ms to 0.75 s. This modulation is attributed to the changing electron diffusion coefficient, which is influenced by the exponential trap distribution. Leveraging these characteristics, we demonstrated that synaptic features could be precisely controlled by varying the intensity of the incident light, achieving zero power consumption. To verify the feasibility of PRC using DSC-based synaptic devices, we conducted time-series data processing tasks, such as STM and PC tasks, using various pulse widths and light intensities. Both *C*_STM_ and *C*_PC_ reached 1.31 and 1.13, respectively. This finding indicates that these devices are photovoltaic-based artificial synapse devices that can be driven by incident light input signals. Additionally, we observed that high *C* values can be achieved at *P* values at which τ_rise_ and *T*_p_ are comparable. Our findings also reveal that the operating time scale of PRC with DSC-based synaptic devices can be altered by adjusting the *P*. Furthermore, the device demonstrated a high accuracy of 92% in motion recognition tasks. This study represents the first time that the potential application of self-powered artificial synapses with solar cells to PRC has been investigated. Our findings pave the way for the realization of multiple-time scale PRC, highlighting the potential for advanced applications in edge AI and neuromorphic computing.

## Experimental Section

### Device Fabrication

A fluorine-doped tin oxide (FTO) glass substrate (size: 2.5 × 2.5 cm, conductivity: 7 Ω/□, NPV-CFT2–7C, AS ONE CORPORATION, Japan) was cleaned with acetone, ethanol, and deionized water. Then, TiO_2_ paste (PST-18NR, JGC Catalysts and Chemicals Ltd., Japan) was coated onto the FTO glass substrate using the doctor blade method. Then, the TiO_2_ electrode was heated at 150 °C for 30 min, followed by heating at 450 °C for 30 min. Typically, the thickness of the TiO_2_ layer was 3 μm. The TiO_2_ electrode was immersed in a 0.1 mM squarylium derivative-based dye (SQ2, Solaronix S.A., Aubonne, Switzerland) solution in acetonitrile (014-00386, FUJIFILM Wako Pure Chemical Corporation, Japan) for 24 h. Next, the dye-coated TiO_2_ electrode was assembled with a thermoplastic spacer (HIMILAN, Dow-Mitsui Polychemicals Company, Ltd., Japan) and filled with 0.15 M triiodide solution (Z-150, Solaronix S.A., Aubonne, Switzerland) as the electrolyte. Finally, a Pt plate (size: 2 × 2 cm) was assembled as the counter electrode. The active electrode area was typically 1.5 cm^2^. All processes were conducted under ambient pressure and room temperature.

### Device Characterization

IPCE spectra were obtained using a Peccell Technologies S10AC system with a 150 W xenon lamp. We set the step interval to 5 nm and the delay to 2 s. Electrical measurements were performed using a source meter (2400, Keithley, OH, USA), DAQ device (USB-6366, National Instruments, TX, USA), and laser diode (λ = 658 nm, L658P040, THORLABS Inc., USA). The laser spot diameter was less than 4 mm. The radiation power ranged from 0.01 to 15 mW. The time constants were obtained from the transient open-circuit voltage induced by laser irradiation (*T*_p_: 5 s). All processes were conducted under ambient pressure and room temperature.

### Time-Series Data Processing Task

The input waveform consisted of 1000 random binary signals, where 0 and 1 represented the ON and OFF states of the laser irradiation, respectively. *T*_p_ ranged from 1 to 1000 ms. The number of virtual nodes was set to 125. The washout was set to 200 pulses. The technical details have been previously presented elsewhere.^19,40^ We fabricated eight devices and performed STM and PC tasks with two data sets, respectively.

### Motion Recognition Task

Motion recognition data sets were derived from three persons with six actions (i.e., bend, wave1, wave2, jump, run, and side). Bend, wave1, and wave2 represent crunching, waving one hand, and waving both hands, respectively. Jump, run, and side represent actions to move from one edge of the screen to the other by jumping, running, or moving sideways, respectively. Motions were recorded using a commercial camera (EOS Kiss X9, Canon Inc., Japan) at 60 FPS. The video duration varies slightly depending on the type of video. The video was converted into a binary format at 30FPS. Subsequently, the video was divided into eight strips. To convert the video into a time variation of the laser *P*, we calculated the average luminance *P*_ave_(*t*) from *P*(*x*, *y*, *t*), which is the luminance of each pixel in each strip. Then, to highlight the motion features, we calculated . *P*_min_ and *P*_max_ are the minimum and maximum values of *P*(*t*), respectively. The obtained time-series data were fed into the laser, and the transient response of *V*_oc_ was measured. *P*_max_ was set to 1 mW. To preserve the motion speed, *T*_p_ was set to 0.033 s. The readout 0 s was defined as the time at which the video ended. We measured *V*_oc_ at a readout of 10 ms and fed it into a simple NN with one layer. We used an open-source library (Keras) for NN processing. We used cross-entropy loss functions. The NN consists of a 1 × 8 input layer and 1 × 6 output layer and no hidden layers. Softmax function and RMSprop were used as activation functions and optimizer, respectively. The video database consisted of 24 videos per person in one action. We used 57 videos per action for training and 15 videos for testing.

## Data Availability

The data sets used and/or analyzed during this study are available from the corresponding author on reasonable request.

## Supporting Information Available

Transient response of polycrystalline Si solar cells, rise time constant of Si solar cells and DSC as a function of *P*, result of time-series processing task with and without DSC-based synaptic device, motion recognition accuracy with various machine learning algorithms, and motion recognition accuracy with and without DSC-based synaptic devices (PDF)

## Abbreviations

AI: artificial intelligence
ANN: artificial neural network
DSC: dye-sensitized solar cell
FTO: fluorine-doped tin oxide
IPCE: incident photon-to-current conversion efficiency
MT: multiple trapping
PC: parity check
PPD: paired pulse depression
PPF: paired pulse facilitation
PRC: physical reservoir computing
RC: reservoir computing
STM: short-term memory
STP: short-term plasticity
