# Supplementary material for: Self-Powered Dye-Sensitized Solar-Cell-Based Synaptic Devices for Multi-Scale Time-Series Data Processing in Physical Reservoir Computing
Source: ACS Appl Mater Interfaces. 2024 Oct 28;17(3):5056–65. doi: 10.1021/acsami.4c11061 (PMC11758776; doi:10.1021/acsami.4c11061)
Supplement: Supplementary file 1 — am4c11061_si_001.pdf [file am4c11061_si_001.pdf]

Supporting Information

Self-Powered Dye-Sensitized Solar Cell-Based Synaptic Devices for Multi-Scale Time-Series Data Processing in Physical Reservoir Computing

*Hiroaki Komatsu, Norika Hosoda, and Takashi Ikuno**

Department of Applied Electronics, Graduate School of Advanced Engineering, Tokyo University of Science, Katsushika, Tokyo 125-8585, Japan

*Email: tikuno@rs.tus.ac.jp

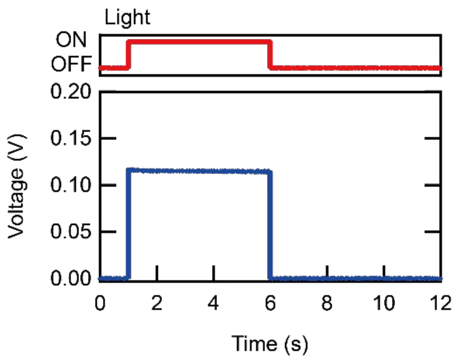

Figure S1. Typical transient voltage response of polycrystalline Si solar cells (λ: 658 nm, T_p: 5 s, P: 5.0 mW).

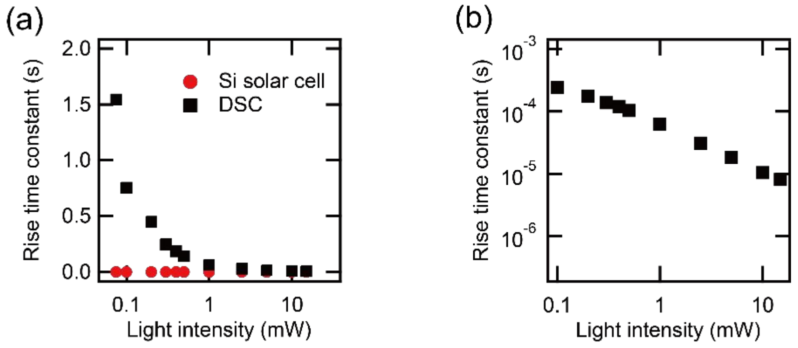

Figure S2. Rise time constant as a function of *P*. (a) Rise time constant for DSC and polycrystalline Si solar cell as a function of *P*. (b) Rise time constant of Si solar cell as a function of *P*.

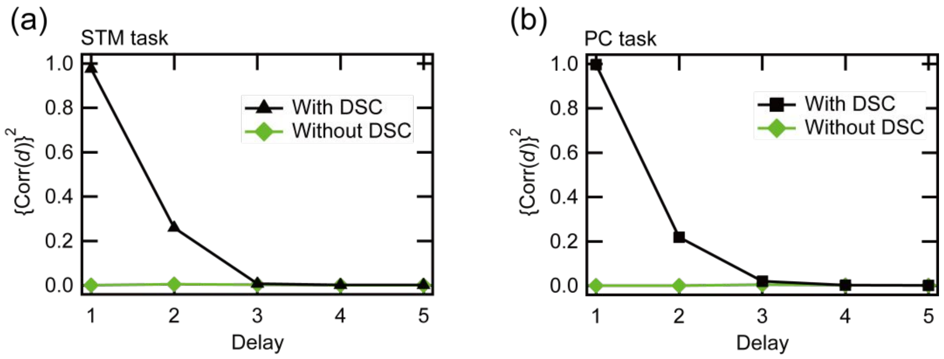

Figure S3. The result of time-series processing task with and without DSC-based synaptic device. (a, b) Typical forgetting curves of the STM and PC tasks with and without DSC-based synaptic device (d: 1, T_p: 100 ms, P: 1 mW, number of virtual nodes: 125).

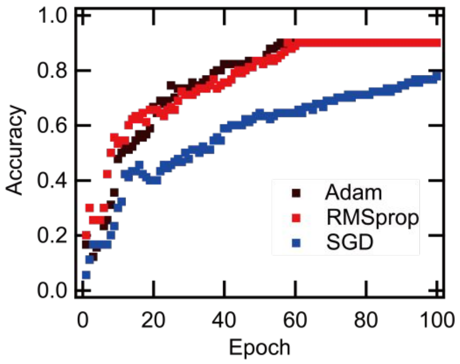

Figure S4. The motion recognition task accuracy as a function of epoch number with various machine learning algorithms.

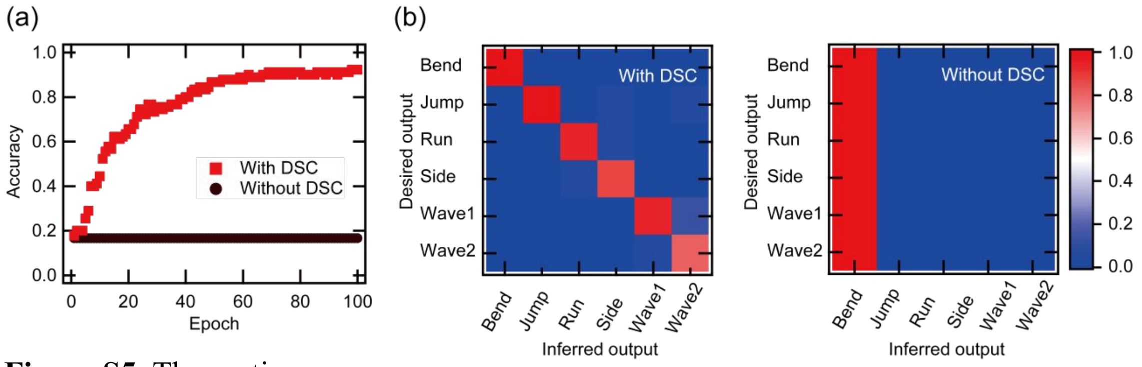

Figure S5. The motion
recognition task result with and without DSC-based synaptic device. (a)Calculated accuracy of the motion recognition task with and without the DSC-based synaptic device. (b) Confusion matrix of motion recognition task with and without DSC-based synaptic device.
